# Erythropoietin Modulates Autophagy Signaling in the Developing Rat Brain in an *In Vivo* Model of Oxygen-Toxicity

**DOI:** 10.3390/ijms131012939

**Published:** 2012-10-10

**Authors:** Ivo Bendix, Corina Schulze, Clarissa von Haefen, Alexandra Gellhaus, Stefanie Endesfelder, Rolf Heumann, Ursula Felderhoff-Mueser, Marco Sifringer

**Affiliations:** 1 Department of Pediatrics I, Neonatology, University Hospital Essen, 45122 Essen, Germany; E-Mail: ursula.felderhoff@uk-essen.de; 2 Department of Anaesthesiology and Intensive Care Medicine, Charité-Universitätsmedizin Berlin, Campus Virchow-Klinikum, 13353 Berlin, Germany; E-Mails: corina_schulze@gmx.de (C.S.); clarissa.von-haefen@charite.de (C.H.); marco.sifringer@charite.de (M.S.); 3 Institute of Molecular Biology, University of Duisburg-Essen, 45122 Essen, Germany; E-Mail: alexandra.gellhaus@uk-essen.de; 4 Department of Neonatology, Charité-Universitätsmedizin Berlin, Campus Virchow-Klinikum, 13353 Berlin, Germany; E-Mail: stefanie.endesfelder@charite.de; 5 Department of Molecular Neurobiochemistry, Ruhr-University Bochum, 44780 Bochum, Germany; E-Mail: rolf.heumann@rub.de

**Keywords:** autophagym, developing brain, oxidative stress, hyperoxia, erythropoietin

## Abstract

Autophagy is a self-degradative process that involves turnover and recycling of cytoplasmic components in healthy and diseased tissue. Autophagy has been shown to be protective at the early stages of programmed cell death but it can also promote apoptosis under certain conditions. Earlier we demonstrated that oxygen contributes to the pathogenesis of neonatal brain damage, which can be ameliorated by intervention with recombinant human erythropoietin (rhEpo). Extrinsic- and intrinsic apoptotic pathways are involved in oxygen induced neurotoxicity but the role of autophagy in this model is unclear. We analyzed the expression of autophagy activity markers in the immature rodent brain after exposure to elevated oxygen concentrations. We observed a hyperoxia-exposure dependent regulation of autophagy-related gene (Atg) proteins Atg3, 5, 12, Beclin-1, microtubule-associated protein 1 light chain 3 (LC3), LC3A-II, and LC3B-II which are all key autophagy activity proteins. Interestingly, a single injection with rhEpo at the onset of hyperoxia counteracted these oxygen-mediated effects. Our results indicate that rhEpo generates its protective effect by modifying the key autophagy activity proteins.

## 1. Introduction

Toxic levels of oxidative stress and reactive oxygen species (ROS) are a common pathway in many neurodegenerative disorders. ROS can induce widespread damage in neuronal cells which can be prevented by activation of antioxidant responses including autophagy [1]. Autophagy is a complex cellular process that involves digestion and recycling of intracellular components via a lysosomal pathway [2–4]. It is a multi-step process that depends on the selective combination of autophagy-related gene (Atg) proteins involving the formation of double-membrane vesicles known as autophagosomes. These autophagosomes mature and fuse with lysosomes and their contents are degraded in an environment mediated by hydrolases [2,5,6].

Proteins of autophagy activity include several Atgs recruited for the formation of the autophagosome [7]. Proteins well described in this process are Beclin-1 and microtubule-associated protein 1 light chain 3 (LC3). Beclin-1, a Bcl-2-homology (BH)-3 domain only protein [8], is important for localization of autophagic proteins to a pre-autophagosomal structure and for mediating the nucleation process [9]. It is distributed within the plasma-membrane, cytoplasm and nucleus, and has the capability to self-associate [10]. Oligomerized Beclin-1 has been proposed to build a platform for rapid nucleation of its associating proteins [9]. Moreover, Beclin-1 and various Atgs form autophagy-inducing complexes at different stages and play an essential role in autophagosome formation [11,12]. Atgs, like Atg3, 5 and 12, are known to be involved in the initiation of autophagy by vesicle expansion and completion [5]. It has been shown that autophagy depends on Atg5/Atg7 and is associated with LC3 truncation [9]. Lacking *Atg5* or *Atg7* induces cell death, the accumulation of damaged ubiquitinated proteins and causes premature lethality [13–15]. Conjugation reactions of Atg12 and LC3 are catalyzed by Atg10 and Atg3, whereby a second conjugation of LC3 to phosphatidylethanolamine and the subsequent formation of autophagosome are dependent on the Atg12-Atg5-Atg16L complex [16]. After activation of autophagy LC3-I is conjugated with phosphatidylethanolamine and then converted to membrane-bound LC3-II [17]. Thereby, lipidated LC3-II is one of the main components of the autophagosome membrane, forming a suitable marker for monitoring autophagosome formation and autophagic cell death [18].

Various mechanisms have been discovered to induce autophagy, with ROS being essential for autophagy activation. Oxidative stress triggers autophagy as an active response to degrade and recycle damaged cellular material [6,19,20]. Furthermore, autophagy has been shown to be protective in stressful conditions and might be involved in preventing neuro-degeneration in the adult brain [13,14,21–23]. However, autophagy has also been described as a prominent feature of cell death during embryonic development. It displays a specific type of programmed cell death, suggesting that autophagic (type II) death may be distinct from apoptotic (type I) death [24–26]. Additionally, cross-talking between autophagy and apoptosis has been described and autophagy can precede apoptosis. In the early stages of programmed cell death [27] it is found to be protective but it can also promote apoptosis under particular circumstances [28].

We have previously described a hyperoxia-mediated increase of neuro-degeneration and modulation of apoptotic components [29–32]. A question yet to be answered is whether autophagy activity is affected by oxygen in the neonatal rat brain. Due to the neuro-protective ability of rhEpo in different neonatal brain injury models [31,33–36], we also investigated the effect of rhEpo on key autophagy activity proteins in hyperoxia-mediated neonatal brain injury.

## 2. Results and Discussion

### 2.1. Results

#### 2.1.1. Erythropoietin Ameliorates Hyperoxia-Induced Changes of Beclin-1

**Figure 1:**
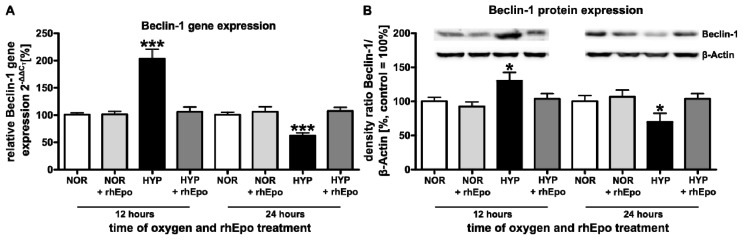
rhEpo restores hyperoxia-triggered changes of Beclin-1 in the developing brain. (**A**) Quantitative analysis of *Beclin-1* mRNA expression by real-time polymerase chain reaction (PCR) after 12 or 24 h of hyperoxia with or without rhEpo treatment. (**B**) Beclin-1 protein expression after 12 or 24 h of hyperoxia with or without rhEpo treatment; the densitometric data represent the ratio of the pixel intensity of the Beclin-1 band to the corresponding β-actin band. Blots are representative of a series of three blots. White bar normoxic control (NOR); light grey bar normoxic control + rhEpo; black bar hyperoxia (HYP); dark grey bar hyperoxia + rhEpo. Data are normalized to levels of rat pups exposed to normoxia (control 100%; bars represent mean + SEM, *n* = 8 per group, *** *p* < 0.001, * *p* < 0.05, independent *t*-test after one-way ANOVA, bonferroni corrected compared to respective controls).

As Beclin-1 is one of the key players in autophagy induction and can intervene at almost every step of the autophagic process [9], we investigated the oxygen and rhEpo mediated regulation of Beclin-1. Quantitative analysis of mRNA expression by real-time PCR (Figure 1A) showed a marked up-regulation of *Beclin-1* mRNA expression in brain hemispheres of rat pups after 12 h (203.1 + 18.0%) and a significant down-regulation after 24 h (62.1 + 5.4%) of hyperoxia (black bars). A single rhEpo-injection ameliorated this effect (dark grey bars) to normoxia controls (white bars). Analysis of Beclin-1 protein expression assessed by Western blot was in accordance with the real-time PCR results. Protein expression of Beclin-1 was significantly increased after 12 h (130.3 + 12.4%) and decreased after 24 h (69.7 + 12.8%); a single application of rhEpo normalized Beclin-1 protein expression to the control level (Figure 1B).

#### 2.1.2. Intervention with rhEpo Modifies Oxygen Triggered Alterations of Autophagy-Related Components

Considering that autophagy is a complex multistep process involving several Atg proteins [6], we investigated the regulation of specific Atg-members known to be involved in the process of autophagosome formation.

**Figure 2:**
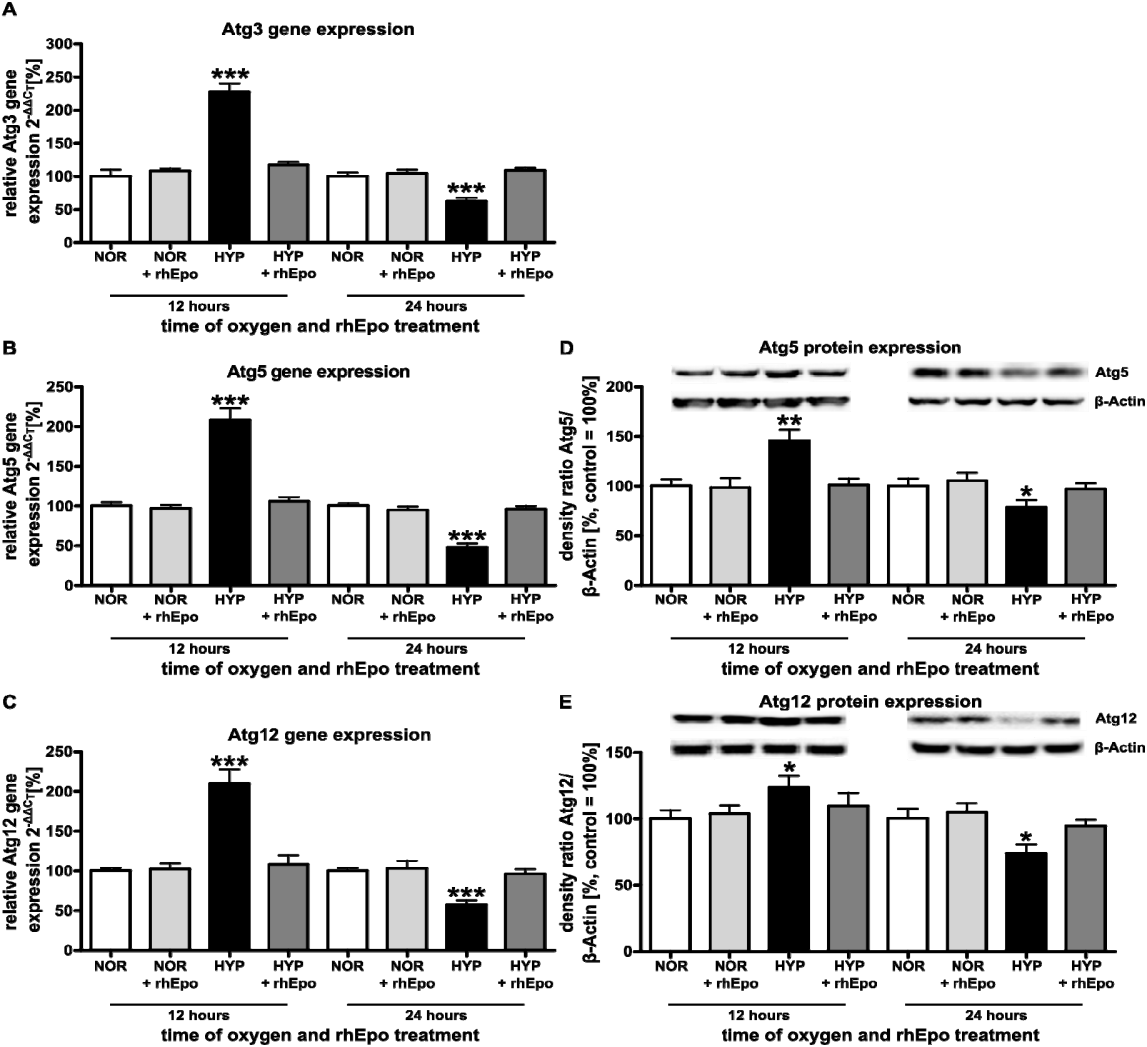
Hyperoxia-triggered alterations of autophagy-related components are modified by rhEpo treatment. (**A**–**C**) Quantitative analysis of *Atg3*, *5* and *12* mRNA expression after 12 h and 24 h of high oxygen concentration with or without rhEpo treatment. (**D**,**E**) Atg5 and Atg12 protein expression, densitometric data represent the ratio of the pixel intensity of the Atg5 or Atg12-Atg5 complex band to the corresponding β-actin band. White bar normoxic control (NOR); light grey bar normoxic control + rhEpo; black bar hyperoxia (HYP); dark grey bar hyperoxia + rhEpo. Data are normalized to levels of rat pups exposed to normoxia (control 100%; bars represent mean + SEM, *n* = 8 per group, *** *p* < 0.001, ** *p* < 0.01, * *p* < 0.05, independent *t*-test after one-way ANOVA, bonferroni corrected compared to respective controls).

Analysis of mRNA expression revealed a distinct up-regulation of *Atg3*, *5 and 12* in the brain hemispheres of rat pups after 12 h (*Atg3*: 227.1 + 13.0%; *Atg5*: 207.7 + 15.6%; *Atg12*: 209.6 + 18.0%) and a significant down-regulation after 24 h (*Atg3*: 62.4 + 5.3%; *Atg5*: 48.0 + 4.9%; *Atg12*: 57.2 + 5.9%) of exposure to high oxygen concentrations (Figure 2A–C). Analysis of Atg5 and Atg12 protein expression was in accordance with mRNA expression. Both Atg proteins were significantly increased after 12 h (Atg5: 145.7 + 11.2%; Atg12: 123.7 + 8.6%) and decreased after 24 h (Atg5: 78.5 + 7.5%; Atg12: 73.9 + 7.0%). A single application of rhEpo diminished oxygen-induced impairment of Atg5 and Atg12 expression to control levels (Figure 2D–E).

#### 2.1.3. Erythropoietin Restores Hyperoxia-Mediated Changes of LC3A-II and LC3B-II in the Developing Brain

**Figure 3:**
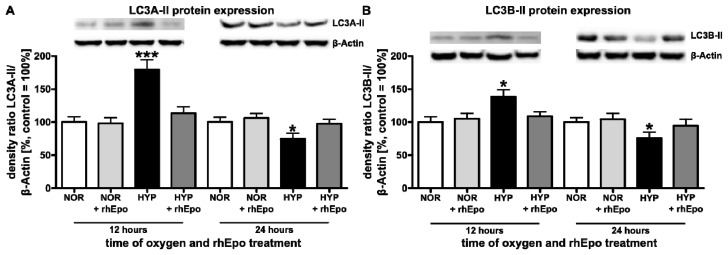
Erythropoietin restores hyperoxia–mediated changes of LC3A-II and LC3B-II in the developing brain. (**A**,**B**) LC3A-II and LC3B-II protein expression, the densitometric data represent the ratio of the pixel intensity of the LC3A-II or LC3B-II band to the corresponding β-actin band. White bar normoxic control (NOR); light grey bar normoxic control + rhEpo; black bar hyperoxia (HYP); dark grey bar hyperoxia + rhEpo. Data are normalized to levels of rat pups exposed to normoxia (control 100%; bars represent mean + SEM, *n* = 8 per group, *** *p* < 0.001, * *p* < 0.05, independent *t*-test after one-way ANOVA, bonferroni corrected compared to respective controls).

On account of the processing of LC3 proteins involved in the formation and elongation of the autophagosome [20], we further investigated the effect of hyperoxia and concomitant rhEpo-treatment in the developing rat brain. As revealed by Western blot analysis (Figure 3), expression of the type II form of LC3A and LC3B displays a significant up-regulation after 12 h (LC3A-II: 179.2 + 15.1%; LC3B-II: 138.1 + 10.9%) and a significant down-regulation after 24 h (LC3A-II: 74.7 + 8.7%; LC3B-II: 75.6 + 9.3%) of hyperoxia. However, a single application of rhEpo restores expression of both LC3 proteins to normoxic control levels.

### 2.2. Discussion

Our study demonstrates that high oxygen conditions lead to a modification of markers for autophagic cell death in the developing brain. Moreover, we showed that a single rhEpo-treatment neutralizes these modifications. In previous studies, our group demonstrated a marked reduction of inflammatory mediators, oxidative stress, apoptotic cell death and pro-apoptotic factors in immature rodent brains exposed to hyperoxia after rhEpo treatment [31,37,38].

As demonstrated by several groups, oxidative stress culminating in ROS generation is believed to be essential to trigger autophagy by various mechanisms [19,39]. Once autophagy is activated the formation of phagophores (precursors of autophagosomes) is initiated by Beclin-1 [40]. Here, we show that hyperoxia modulates Beclin-1 expression in the immature brain resulting in an up-regulation after 12 h of hyperoxia. In line with this finding, previous reports revealed that oxidative stress-induced autophagy coincides with an increase in the level of Beclin-1 in cortical neurons, glioma U251 and neuronal PC12 cells [41–43]. The elongation of phagophores requires two ubiquitin-like conjugation systems; the Atg12-Atg5-Atg16L and the phosphatidylethanolamine-LC3 system [4,18]. Some Atg genes, including LC3, were found to be up-regulated during oxidative stress-induced autophagy induction, suggesting a possible function for ROS in regulation of Atg protein expression [44]. Noteworthy, in our model of oxygen toxicity we demonstrated an increased expression of Atg3, Atg5, Atg12, LC3A-II and LC3B-II after 12 h of oxygen exposure suggesting that hyperoxia induced oxidative stress in the immature brain might trigger these acute changes in autophagy activity.

Studies using mutant mice with targeted deletion of *Atg5* or *Atg7* specifically in the brain suggested that autophagy is constitutively active and essential for neuronal survival [13–15]. However, these studies analyzed the function of autophagy activity proteins in the healthy adult brain and used a constitutive knock out model neglecting the dynamic changes of autophagic processes and their relevance in different pathological conditions. In contrast, we were interested in the regulation of autophagy activity in the immature brain and applied different levels of oxidative stress by varying the duration of hyperoxia. This might have led to an altered tissue environment with modified inflammatory mediators [38]. These different experimental setups might explain the discrepancy between hyperoxia induced brain damage and enhanced autophagy activity. Interestingly the autophagic response seems to depend on the duration of oxygen exposure, since we observed a marked down-regulation of all autophagy related proteins analyzed after 24 h.

In previous studies we demonstrated an increase in apoptotic cell death present after 6 h of hyperoxia and evident up to 72 h [29,30]. The dynamic changes of apoptotic processes combined with the regulation of autophagic activity shown here support the hypothesis of an interaction between these two mechanisms. This indicates an induction of autophagy by hyperoxia-triggered acute ROS formation with enhanced activation of apoptosis as previously shown [28]. However, with sustained oxygen exposure, triggering both extrinsic and intrinsic apoptotic cascades [29,32], autophagy might be inhibited by apoptosis [28]. In this regard it has been shown that caspase-2 deficiency leads to increased autophagy and prolonged neuronal survival whereas mitochondrial oxidative stress induced apoptosis is inhibited [45]. Of note, we have recently demonstrated that the caspase-2 initiated intrinsic apoptotic pathway plays a major role in hyperoxia-induced cell death and might be a potential explanation for the observed increased apoptosis [32] as well as reduced autophagy after longer durations of hyperoxia. This might be highly relevant for the resulting damage to the developing brain and long term functional outcome [46], since the injury cascade is in full progress within this critical time window and autophagy is necessary to degrade and recycle damaged cellular material [6,19,20].

Interestingly rhEpo reverses these hyperoxia mediated effects. However, whether these rhEpo triggered modulations are primary or secondary effects remains to be clarified. One possible mechanism would be amelioration of ROS-formation by rhEpo [36,37] indirectly inhibiting oxygen induced autophagy within the early stage (12 h). When apoptosis is predominant at 24 h it might induce anti-apoptotic signaling thereby enabling autophagy. Another explanation could be a direct modulating process on autophagy by stabilization of the outer mitochondrial membrane mediated by Bcl-2 stabilization. This would lead to impaired permeabilization of the outer mitochondrial membrane or directly via an interaction of Bcl-2 with Beclin-1 [36,47].

Although we indicate that autophagy is involved in hyperoxia mediated brain damage, we cannot exclude that our results are partly associated with apoptosis, since it has been reported that e.g. Beclin-1 may have dual roles, in apoptosis and autophagy [41,48]. Knockout mice deficient in *Beclin-1* show both, neuro-degeneration and lysosomal abnormalities [49]. Since the autophagic process is dependent on the interaction of Beclin-1 and the anti-apoptotic protein B-cell lymphoma 2 (Bcl-2), the Beclin-1-Bcl-2 complex functions as a regulator of autophagy depending on the tissue environment and the pathological conditions [50]. Accordingly, we recently demonstrated that hyperoxia triggers a decrease in Bcl-2 expression as a part of the intrinsic apoptotic pathway in the developing brain [32]. This finding might explain the observed modulation of Beclin-1 expression counteracted by rhEpo, giving similar results in a model of traumatic brain injury [36]. Furthermore, it has been shown that Beclin-1 is a substrate of caspase-3, which is a key player in the intrinsic apoptotic pathway. The cleavage products of Beclin-1 reduce autophagy and promoted apoptosis [51].

In order to distinguish to what extent apoptosis and autophagy contribute to hyperoxia mediated cell death further studies are required. Nevertheless, our data suggest that the simultaneous regulation of a broad range of autophagy activity proteins after oxygen exposure play an important role in the pathology of neonatal brain damage.

## 3. Experimental Section

### 3.1. Animals and Experimental Procedure

#### 3.1.1. Exposure to Hyperoxia

Six-day old (P6) Wistar rat pups (BgVV, Berlin, Germany) and their dams were placed into an OxyCycler (BioSpherix, Lacona, NY, USA) maintained at 80% oxygen. Animals were exposed to hyperoxia (FiO_2_ 80% O_2_) or normoxia (FiO_2_ 21%, room-air) for 12 and 24 h, and were sacrificed at the end of exposure.

#### 3.1.2. Treatment Protocols

Sex-matched P6 Wistar rat pups (BgVV) were randomly assigned to four groups and treated as follows: (1) normoxia (NOR, FiO_2_ 21% O_2_) and normal saline IP. injections; (2) normoxia and 20,000 IU/kg rhEpo (NOR + rhEpo, NeoRecormon^®^, Boehringer-La Roche, Grenzach, Germany) IP. injections; (3) hyperoxia (HYP, FiO_2_ 80% O_2_) and normal saline IP. injections; (4) hyperoxia and 20,000 IU/kg rhEpo (HYP + rhEpo) IP. injections. In all study groups rhEpo or normal saline was injected at the beginning of an oxygen/room air exposure.

All procedures were approved by the local state authorities for animal welfare and followed institutional guidelines.

### 3.2. Tissue Sampling

Twelve or 24 h after hyperoxia initiation on P6, Wistar rat pups (*n* = 8 per group) were sacrificed with chloral hydrate (1 mg/kg, IP.) and were transcardially perfused with normal saline solution. After decapitation the olfactory bulb and cerebellum were removed, brain hemispheres were snap-frozen in liquid nitrogen and stored at −80 °C until further analysis (quantitative real-time polymerase chain reaction (PCR) and Western blotting).

### 3.3. Semiquantitative Real-Time PCR

Total cellular RNA was isolated from snap-frozen tissue by acidic phenol/chloroform extraction and DNase I treated (Roche Diagnostics, Mannheim, Germany); 2 μg of RNA was reverse transcribed with Moloney murine leukemia virus reverse transcriptase (Promega, Madison, WI, USA) in 25 μL of reaction mixture. The resulting cDNA (1 μL) was amplified by real-time PCR. The PCR product of *Atg3*, *Atg5*, *Atg12 and Beclin-1* was quantified in real-time, using a dye-labeled fluorogenic reporter oligonucleotide probe and primers (Table 1). All probes were labeled at their 5′ ends with the reporter dye 6-carboxy-fluoresceine (FAM), at their 3′ ends with the quencher dye 6-carboxytetramethylrhodamine (TAMRA) and were purchased from BioTez (Berlin, Germany). Hypoxanthineguanine phosphoribosyl-transferase (HPRT) was used as internal standard. Real-time PCR and detection were performed in triplicate and repeated four times for each sample using a total reactive volume of 13 μL which contained 6.5 μL of 2× TaqMan Universal PCR Master Mix (Applied Biosystems, Foster City, CA, USA), 2.5 μL of 1.25 μM oligonucleotide mix and 0.5 μL (0.5 μM) of probe. The PCR amplification was performed in 96-well optical reaction plates for 40 cycles with each cycle at 94 °C for 15 s and 60 °C for 1 min. Each plate included at least three “no template controls”. The expressions of *Atg3*, *Atg5*, *Atg12*, *Beclin-1* and *HPRT* were analyzed with the real-time PCR ABI Prism 7500 sequence detection system (Applied Biosystems) according to the 2^−ΔΔC_T_^ method [52].

### 3.4. Immunoblotting

Snap-frozen tissue was homogenized in RIPA (radioimmunoprecipitation assay) buffer (1% NP40, 0.5% sodium deoxycholate, 0.1% SDS, 1 mM EDTA, 1 mM EGTA, 1 mM Na_3_VO_4_, 20 mM NaF, 0.5 mM DTT, 1 mM PMSF and protease inhibitor cocktail in PBS pH 7.4). The homogenate was centrifuged at 1050 g (4 °C) for 10 min, and the microsomal fraction was subsequently centrifuged at 17,000 *g* (4 °C) for 20 min. After collecting the supernatant, protein concentrations were determined using the bicinchoninic acid kit (Interchim, Montluçon, France). Protein extracts (25 μg per sample) were denaturated in Laemmli sample loading buffer at 95 °C, separated by 10% or 15% sodium dodecyl sulfate polyacrylamide gel electrophoresis and electro-transferred in transfer buffer to a nitrocellulose membrane (0.2 μm pore, Protran; Schleicher & Schüll, Dassel, Germany). Nonspecific protein binding was prevented by treating the membrane with 5% non-fat dry milk in Tris-buffered saline/0.1% Tween 20 for 2 h at room temperature. Equal loading and transfer of proteins was confirmed by staining the membranes with Ponceau S solution (Fluka, Buchs, Switzerland). The membranes were incubated overnight at 4 °C with rabbit monoclonal anti-Atg5 (55 kDa; 1:1.000; Cell Signaling Technology, Danvers, MA, USA), rabbit monoclonal anti-Atg12 (55 kDa; 1:1.000; Cell Signaling Technology), rabbit monoclonal anti-Beclin-1 (60 kDa; 1:1.000; Cell Signaling Technology), rabbit monoclonal anti-LC3A (14 kDa; 1:500; Cell Signaling Technology) or rabbit monoclonal anti-LC3B (14 kDa; 1:500; Cell Signaling Technology), respectively. Unfortunately we did not get any specific Atg3 signal for three commercial Atg3 antibodies tested. Secondary incubations were performed with horseradish peroxidase-linked anti-rabbit (1:2.000; Amersham Biosciences, Bucks, United Kingdom) antibody. Positive signals were visualized using enhanced chemiluminescence (ECL; Amersham Biosciences) and quantified using a ChemiDoc^™^ XRS+ system and the software Quantity One^®^ (Bio-Rad, Munich, Germany). Membranes were stripped, then washed, blocked and re-probed overnight at 4 °C with mouse anti-β-actin monoclonal antibody (42 kDa; 1:10.000; Sigma-Aldrich, Taufkirchen, Germany).

### 3.5. Statistical Analysis

Data were analyzed using IBM SPSS Statistics (IBM, Frankfurt, Germany) and presented as mean + standard error (SEM). Group effects were assessed by analysis of variance (ANOVA) followed by *post-hoc* independent sample *t*-test multiple comparison. *p*-values are presented after Bonferroni correction. Adjusted *p*-values of <0.05 were considered statistically significant.

## 4. Conclusions

In the present study, we report that the exposure of neonatal rats to high concentrations of oxygen causes modifications in autophagic activity in the immature rat brain. Our results show that a single rhEpo application restores hyperoxia-mediated changes in the levels of the key autophagy proteins Beclin-1, Atg3, 5, 12, LC3A-II and LC3B-II. Our findings indicate an additional mechanism might be responsible for the protective effects of rhEpo in the context of hyperoxia to the developing brain.

## Figures and Tables

**Table 1:** Sequences of oligonucleotides and gene locus. HPRT: Hypoxanthine-guanine phosphoribosyl-transferase; Atg: autophagy-related gene protein.

Gene		Oligonucleotide sequences 5′-3′	
*Atg3*	forward	TGCGACAGTCTCTCCGTGC	NM_0134394
	reverse	GGCCACTTCCAGAGCCTTTC	
	probe	TGCTCCGGTCCCAGGATGCAGA	

*Atg5*	forward	ACATCAGCATTGTGCCCCA	NM_001014250
	reverse	TGTCATGCTTCGGTGTCCTG	
	probe	CAGACTGAAGGCCGTGTCCTGCTCA	

*Atg12*	forward	TCTGCCTAGCCTGGAACTCAG	NM_001038495
	reverse	TAGCCCTGTGTGCTCTGCTTT	
	probe	CCTGTCCGTGAAGCTCACCCAGC	

*Beclin-1*	forward	GCAGCACCATGCAGGTGAG	NM_053739
	reverse	TGGTCACTCGGTCCAGGATC	
	probe	TCGTGTGCCAGCGCTGTAGCCA	

*HPRT*	forward	GGAAAGAACGTCTTGATTGTTGAA	NM_012583
	reverse	CCAACACTTCGAGAGGTCCTTTT	
	probe	CTTTCCTTGGTCAAGCAGTACAGCCCC	
